# Optimized Solid-State Fermentation of Sugar Beet Pulp with Mixed Microbes Improves Its Nutritional Value and Promotes Growth, Health, and Intestinal Function in Yellow Catfish (*Pelteobagrus fulvidraco*)

**DOI:** 10.3390/ani16060915

**Published:** 2026-03-14

**Authors:** Ning Qiu, Tanqing Chi, Xuan Luo, Hao Yang, Chi Zhang, Hongsen Xu, Xin Liu

**Affiliations:** 1 School of Animal Science and Nutritional Engineering, Wuhan Polytechnic University, Wuhan 430070, China; qiu201620769@outlook.com (N.Q.); 15086624226@163.com (H.Y.); zhch@whpu.edu.cn (C.Z.); hsxu1989@163.com (H.X.); 2 Hubei Key Laboratory of Animal Nutrition and Feed Science, Wuhan Polytechnic University, Wuhan 430023, China

**Keywords:** sugar beet pulp, mixed microbial solid-state fermentation, *Pelteobagrus fulvidraco*, growth performance

## Abstract

The rising cost of conventional protein sources such as soybean meal has prompted the search for sustainable and economical alternatives in aquafeeds. Sugar beet pulp (SBP), an abundant by-product of the sugar industry, possesses nutritional potential but is limited by its high fiber and anti-nutritional factors. Solid-state fermentation (SSF) offers a promising approach to enhance its nutritive value and functional properties. This study evaluated the effects of dietary inclusion of mixed microbial solid-state fermented beet pulp (FBP) on the growth, systemic health and intestinal function of juvenile yellow catfish (*Pelteobagrus fulvidraco*). First, orthogonal optimization determined *Lactiplantibacillus plantarum*:*Saccharomycopsis fibuligera*:*Bacillus subtilis* = 1:3:3 as the optimal ratio, significantly improving the nutritional profile of FBP. Based on this optimized FBP, an 8-week feeding trial, five isonitrogenous and isolipidic diets were formulated by replacing 0–12% soybean meal with FBP. The results demonstrated that 9% FBP inclusion yielded optimal growth performance and significantly improved muscle texture. At the systemic level, FBP supplementation reduced serum lipid markers and liver enzyme activities while enhancing antioxidant capacity. At the intestinal level, FBP promoted intestinal health by increasing key digestive enzyme (lipase, trypsin, amylase) activities, stimulating villus development, and improving intestinal antioxidant status. Furthermore, gut microbiota analysis revealed that dietary FBP supplementation significantly modulated intestinal microbial composition, with notable enrichment of genera such as *Leucobacter*. In conclusion, FBP is a multi-functional ingredient that enhances growth, product quality, systemic physiology, and intestinal health in yellow catfish aquaculture. These findings provide a viable strategy for the sustainable utilization of agricultural by-products in aquafeeds.

## Simple Summary

The rising cost of traditional feed ingredients like soybean meal is a major challenge for fish farming. This study explored a way to turn sugar beet pulp—an abundant and inexpensive by-product from sugar production—into a nutritious feed for yellow catfish. We used a special mix of helpful bacteria and yeast to ferment the pulp, which improved its protein content and reduced its tough fibers. When we replaced 9% of the soybean meal in the fish diet with this fermented pulp, the fish grew better, their muscle texture improved, and they showed signs of better overall health, including healthier livers and intestines. The fermented pulp also increased the number of beneficial bacteria in the fish gut. These results show that fermented sugar beet pulp is a promising, cost-effective feed ingredient that can support sustainable aquaculture by making good use of agricultural leftovers, lowering feed costs, and improving fish quality and welfare.

## 1. Introduction

With the rapid development of intensive aquaculture, the diversification and sustainable utilization of feed ingredients have become major concerns in the industry. In this context, the yellow catfish (*Pelteobagrus fulvidraco*) has grown into one of the most commercially valuable freshwater species in China, prized for its tasty flesh, rapid growth, and strong market demand [1,2,3]. However, this fish relies heavily on high-protein feeds [4], and with the climbing costs of conventional protein sources like fish meal and soybean meal, feed expenses have become a key bottleneck for the industry’s sustainable growth [3,5]. Therefore, exploring cost-effective alternative protein resources, particularly through the bioprocessing of agricultural by-products, is of great significance for reducing feed costs and enhancing the economic viability of yellow catfish aquaculture.

Sugar beet pulp (SBP), a major by-product of the sugar industry, is widely available and inexpensive [6]. SBP is notably rich in fermentable carbohydrates such as pectin and fiber, and it also provides a moderate protein content (7–10% crude protein) [7], qualities that position it as a promising feed ingredient [8]. Earlier research supports this potential: inclusion of beet pulp in diets has been shown to enhance intestinal villus structure, fiber digestion, and gut microbiota in certain fish species [9] as well as in terrestrial livestock such as pigs [10]. These findings indicate documented health-promoting effects. Nevertheless, significant challenges remain. As a plant-derived by-product, beet pulp also presents clear challenges. It contains relatively high levels of anti-nutritional factors (ANFs) and crude fiber, and its palatability tends to be low. Together, these factors have limited its practical application in aquafeeds [11]. To overcome these limitations, bioprocessing techniques are needed to improve its nutritional quality and digestibility.

Among various processing methods, solid-state fermentation (SSF) has been extensively demonstrated to effectively improve the nutritional value and bioavailability of plant by-products [12,13,14]. This process involves microbial secretion of various hydrolytic enzymes that degrade fibers and ANFs, increase crude protein and small peptide content, and promote the formation of functional metabolites [14,15,16]. Notably, these mechanisms are particularly suitable for addressing the high fiber and ANF content inherent in SBP, making SSF an ideal approach for upgrading this by-product. In aquaculture, solid-state or mixed microbial fermentation using lactic acid bacteria, Bacillus, and yeast has been applied to soybean meal [17], cottonseed meal [18], and commercial feeds [19], showing positive effects on growth, antioxidant capacity [20], immunity, and gut structure/microbiota in fish [21,22]. Despite these promising advances, systematic research on the partial replacement of soybean meal with mixed microbial solid-state fermented beet pulp in yellow catfish diets remains limited. Specifically, comprehensive evaluations of its effects on growth performance, muscle quality, serum biochemical and antioxidant parameters, intestinal morphology, and microbial community are lacking.

To address this research gap, this study employed a mixed microbial consortium composed of *Saccharomycopsis fibuligera*, *Lactiplantibacillus plantarum*, and *Bacillus subtilis* for the solid-state fermentation of beet pulp. The rationale for selecting these specific strains was based on their complementary and synergistic functional properties. *Saccharomycopsis fibuligera* was selected for its ability to secrete amylolytic enzymes that break down complex carbohydrates into simpler sugars, thereby providing a readily utilizable carbon source for the other microorganisms while simultaneously synthesizing beneficial compounds such as B group vitamins, amino acids, nucleotides, and the immunostimulatory polysaccharide β-glucan. Its activity also contributes to reducing antinutritional factors and improving palatability and protein utilization [23,24]. *Lactiplantibacillus plantarum* serves as a key probiotic that rapidly ferments available sugars to produce organic acids, lowering the pH to inhibit pathogenic and spoilage microorganisms. Additionally, it degrades fibers to enhance digestibility and modulates the gut microbiota to promote beneficial bacterial proliferation in the host animal [25,26]. *Bacillus subtilis* complements these functions by secreting a diverse array of hydrolytic enzymes—including proteases, cellulases, and xylanases—that break down complex macromolecules and residual antinutritional factors present in the beet pulp. Through competitive exclusion, it inhibits harmful microorganisms, while its enzymatic activities may generate bioactive peptides that enhance immune responses and disease resistance [27,28,29]. In combination, these three microorganisms form a synergistic team: the yeast creates a more accessible substrate, the lactic acid bacterium ensures a favorable fermentation environment through acidification, and the Bacillus provides potent enzymatic capabilities for degrading otherwise indigestible components.

Building upon this synergistic microbial consortium, this study employed solid-state fermentation of beet pulp using *Lactiplantibacillus plantarum*, *Saccharomycopsis fibuligera*, and *Bacillus subtilis*. The fermented product was used to partially replace soybean meal at varying inclusion levels in the diets for yellow catfish. We systematically evaluated its effects on growth performance, muscle quality, serum biochemistry, antioxidant status, intestinal histomorphology, and gut microbiota composition. The objectives of this research were to elucidate the nutritional and functional benefits of mixed microbial fermented beet pulp and provide a scientific basis for its value-added utilization and the development of environmentally friendly feeds for yellow catfish aquaculture.

## 2. Materials and Methods

### 2.1. Experimental Fish and Sugar Beet Pulp

Healthy juvenile yellow catfish (*Pelteobagrus fulvidraco*, *n* = 500) were procured from a commercial fish farm located in Jiangxia District, Wuhan, China, and transported to the Aquaculture Laboratory at the East Campus of Wuhan Polytechnic University. Prior to the trial, fish were disinfected with 0.5% povidone-iodine solution at a dosage of 10 mL/L for 5 min to effectively eliminate pathogenic microorganisms on the body surface. Individuals of uniform size and health were selected for the experiment and acclimatized in temporary rearing tanks (2.5 m × 3.0 m × 1.5 m) for a period of two weeks. After acclimatization, fish with an average body weight of approximately 3.56 ± 0.03 g were randomly distributed into five dietary groups (90 fish per group, 3 replicates × 30 fish per tank) using a single-factor completely randomized design.

Sugar beet pulp was obtained from Xiongxian Chenghua Trading Co., Ltd. (Xiongxian, Baoding, China). The SBP was subjected to a sieve analysis, with the resultant powder being passed through an 80-mesh sieve. The experimental microbial strains, *Lactiplantibacillus plantarum*, *Bacillus subtilis*, and *Saccharomycopsis fibuligera*, were provided by the China Center of Industrial Culture Collection (CICC, Beijing, China).

### 2.2. Inoculum Preparation

Glycerol-preserved strains stored at −80 °C were revived by streaking onto respective solid media. *Saccharomycopsis fibuligera*, *Lactiplantibacillus plantarum*, and *Bacillus subtilis* were inoculated onto YPD solid medium (20 g/L peptone, 10 g/L yeast extract, 20 g/L glucose, 20 g/L agar), MRS solid medium (52.2 g/L MRS broth, 20 g/L agar), and LB solid medium (10 g/L tryptone, 5 g/L yeast extract, 10 g/L sodium chloride, 20 g/L agar), respectively, and incubated at 30 °C for 16 h. Single colonies were then selected and transferred to corresponding liquid media. *Saccharomycopsis fibuligera* and *Bacillus subtilis* were subjected to a 16 h culture period at 30 °C, with shaking at 150 rpm, and *Lactiplantibacillus plantarum* was subjected to a 16 h static incubation period at 30 °C. To ensure strain viability, single colonies of *Saccharomycopsis fibuligera* and *Lactiplantibacillus plantarum* were subcultured once in liquid medium.

### 2.3. Orthogonal Experimental Design

An orthogonal experimental design was employed to optimize the mixed microbial inoculum ratio, as this design enables efficient evaluation of multiple factors at different levels with minimal experimental runs, and allows identification of optimal factor combinations and assessment of factor significance through range analysis. Briefly, thirty grams of ground and sieved sugar beet pulp powder were weighed and transferred into a sterile 500 mL conical flask. Sterile water was added to achieve a 1:1 (*w*/*v*) ratio of solid to water. The flask was sealed, wrapped with aluminum foil, autoclaved at 121 °C for 15 min, and cooled. The inoculum volumes of *Lactiplantibacillus plantarum* (A), *Saccharomycopsis fibuligera* (B), and *Bacillus subtilis* (C) served as the experimental variables. The neutral detergent fiber (NDF) degradation rate and crude protein (CP) increase rate were used as evaluation indices, integrated into a comprehensive score D calculated as D = 0.5 × NDF degradation rate + 0.5 × CP increase rate. Fermentation was conducted at 30 °C for 3 days with manual shaking every 12 h to maintain aerobic conditions. Details of the orthogonal experimental design are shown in Table 1.

### 2.4. Preparation of the Experimental Diet

In accordance with the guidelines established by the National Research Council (NRC), a series of five experimental diets were formulated to ensure equivalent nitrogen and fat content, with the objective of meeting the nutritional requirements of yellow catfish. Fermented sugar beet pulp (FBP) was used to replace soybean meal at inclusion levels of 0% (control), 3% (RM3), 6% (RM6), 9% (RM9), and 12% (RM12). To ensure the uniformity of experimental variables and obtain reliable data, soy protein isolate was supplemented to balance dietary protein content, ensuring consistent crude protein and lipid levels across all diets. The basal diet contained high-quality fish meal, soybean meal, corn gluten meal, fish oil, and vitamin-mineral premixes, all procured from certified suppliers and complying with national feed hygiene standards. Dry ingredients were ground, accurately weighed, and mixed thoroughly using a stepwise enlargement procedure. After oil addition, the mixture was pelleted using a laboratory pellet mill. Pellets were air-dried in a cool, ventilated environment, packaged, and stored at −5 °C until use. Proximate composition was periodically verified to ensure dietary consistency throughout the trial. The composition of ingredients and proximate analysis of experimental diets are presented in Table 2.

The feeding trial was conducted for 8 weeks in a recirculating aquaculture system. Yellow catfish were randomly distributed into 15 indoor tanks (300 L capacity, 200 L water volume), with 30 fish per tank. Each diet was assigned to three replicate tanks in a completely randomized design. Throughout the experimental period, water quality parameters were maintained within optimal ranges: temperature 24–28 °C, dissolved oxygen 7.2 ± 0.4 mg/L, ammonia nitrogen < 0.5 mg/L, pH 7.6 ± 0.3, and nitrite < 0.1 mg/L. These parameters were determined using a YSI Pro2030 multi-parameter meter (Yellow Springs Instruments, Yellow Springs, OH, USA), a PHS-3C pH meter (Shanghai Leici Instrument Factory, Shanghai, China), and commercial spectrophotometric assay kits (Nanjing Jiancheng Bioengineering Institute, Nanjing, China), respectively. The fish were fed the experimental diets formulated as shown in Table 2 twice daily at 08:30 and 18:30, and feeding level was 3% to 5% of body weight, according to Huang et al. [30]. The uneaten feed and feces were removed on a daily basis, and a third of the tank water was exchanged at regular intervals. Fish behavior, feeding activity, and health status were monitored daily, and mortality was recorded.

### 2.5. Chemical Analysis of Samples Before and After Fermentation

Representative sampling of beet pulp biomass was performed using the coning and quartering method according to ISO 6497:2002 [31] standards for animal feeding stuffs. The orthogonal experiment for optimizing the mixed strain ratio employed the crude protein increase rate and neutral detergent fiber (NDF) degradation rate as evaluation indices, and selected the fermented sugar beet pulp powder with the highest weighted average score [32,33]. All samples were subjected to desiccation at 45 °C for 5 h, subsequently ground, sieved, and employed as dry samples for analysis. The analytical components included crude protein, crude fiber, crude ash, amino acids, and organic acids. Samples were analyzed for their crude protein (CP), crude fiber (CF), crude ash content according to AOAC [34]. The crude protein was determined using the regular Kjeldahl method (Kjeltec^TM^ 8400; Foss Inc., Hoganas, Sweden). The crude lipid was determined using the Soxhlet method with petroleum ether extraction. Ash was determined by incineration at 550 °C for 16 h. Neutral detergent fiber (NDF) degradation rate was determined using the filter bag technique via an ANKOM 200 Fiber Analyzer (Ankom Technology Corp., Macedon, NY, USA) according to the procedures described by Van Soest et al. [35]. Amino acid composition was determined according to the method reported by Dai et al. [36], while the content of organic acids was measured in both unfermented samples and selected fermented samples following the method described by Miluscia Arnetoli et al. [37].

### 2.6. Sample Collection

At the termination of the feeding trial, all surviving fish were fasted for 24 h, following which their body length and body weight were measured and the fish enumerated. Fifteen fish were randomly selected from each tank, and blood samples were collected from the caudal vein using a 1.5 mL sterile syringe. The serum was prepared in accordance with the method outlined by Liao et al. for subsequent determination of serum biochemical parameters [38]. Following blood collection, fish were dissected to determine individual viscera and liver weights, from which growth performance indices were calculated. The intestine was excised. The foregut was placed in centrifuge tubes and immediately stored at −80 °C for analysis of digestive and antioxidant enzyme activities and 16S rRNA sequencing; the remaining foregut samples were fixed in 4% paraformaldehyde for 24 h, subsequently rinsed with 70% ethanol (2–3 washes), and then transferred to 70% ethanol for preservation to maintain tissue integrity for subsequent morphological analysis. Muscle samples were obtained from the dorsal musculature above the lateral line of three fish per tank for texture profile analysis.

### 2.7. Growth Parameters and Morphological Index

Actual feed intake was calculated by weighing the feed offered prior to feeding and collecting uneaten feed 1 h post-feeding, which was then dried at 60 °C to constant weight. Individual body weight and length were measured to calculate growth and morphological indices, including weight gain rate (WGR), specific growth rate (SGR), feed conversion ratio (FCR), survival rate (SR), condition factor (CF), viscerosomatic index (VSI), and hepatosomatic index (HSI), using the following formulas [39,40,41,42]:

WGR% = [final weight (g) − initial weight (g)] × 100/initial weight (g);

SGR% = [(Ln final weight − Ln initial weight) × 100/56 days];

FCR = total feed intake (g)/[final body weight (g) − initial body weight (g)];

SR (%) = final fish number × 100/initial fish number;

CF (g/cm^3^) = individual body weight (g) × 100/individual body length (cm)^3^;

VSI% = viscera weight × 100/body weight;

HSI% = hepatopancreas weight × 100/body weight;

#### Muscle Texture Analysis

Muscle texture parameters were measured using a TVT-300XP texture analyzer (Perten Instruments, Beijing, China) and a TA-XT2i texture analyzer (Stable Micro Systems, Surrey, UK) [43]. All muscle samples were uniformly bisected into blocks with dimensions of 1.00 cm × 1.00 cm × 0.50 cm (length × width × height). Texture Profile Analysis (TPA) was conducted to determine key parameters. The instrument settings were configured as follows: a pre-test speed of 2 mm/s, a test speed of 2 mm/s, a post-test speed of 2 mm/s, and a deformation of 20% based on sample thickness. Each sample was then exposed to two compression cycles, with an interval of 15 s between each cycle. The subsequent parameters were determined by obtaining the force-time curve: springiness, hardness, cohesiveness, chewiness, adhesiveness, and resilience. Gumminess and chewiness values were calculated by multiplying the hardness and cohesiveness values and the gumminess and springiness values, respectively [44].

### 2.8. Determination of Serum Biochemical Indices

Upon completion of the feeding trial, the fish were fasted for 24 h. Ten representative fish were randomly selected from each aquarium, anesthetized and blood samples were collected from the caudal vein. After clotting at room temperature for 4 h, the serum was obtained by centrifugation (1342× *g* for 10 min at 4 °C) and stored at −80 °C for subsequent analysis. Serum was prepared according to the method described by Niu et al. [45]. The biochemical indices present in the serum were determined in accordance with the method outlined by Shi et al. [46]. The parameters of interest, encompassing aspartate aminotransferase (AST), alanine aminotransferase (ALT), triglyceride (TG), glucose (GLU), total cholesterol (T-CHO), total protein (TP), malondialdehyde (MDA), and catalase (CAT), were measured in accordance with the protocols stipulated by the manufacturer (Nanjing Jiancheng Biotechnology Co., Ltd., Nanjing, China).

### 2.9. Intestinal Digestive Enzyme Activities

The intestinal tissue (0.1 g) that had been thawed was then homogenized with normal saline (1:9 *w*/*v*) in an ice bath. The mixture was subsequently subjected to centrifugation at 2500 r/min for 10 min at 4 °C, with the aim of determining the activity of intestinal digestive enzymes. The supernatant was then analysed [47]. According to the information provided in the kit’s description, trypsin exhibits catalytic activity for the hydrolysis of the ester bond in the substrate arginine ethyl ester at pH 8.0 and 37 °C. Enzyme activity is quantified by monitoring the increase in absorbance at 253 nm. One unit of activity (U/mg protein) is defined as the amount of enzyme per milligram of protein that produces an absorbance change of 0.003 per minute. A blue complex is generated upon the reaction of iodine solution with starch. Amylase, a digestive enzyme found in the saliva of many organisms, can hydrolyze starch. Activity of amylase is therefore measured by comparing the change in light absorption. One unit of amylase activity corresponds to the hydrolysis of 10 mg of starch per milligram of tissue protein under the specified assay conditions (37 °C, 30 min) [48]. The activity of lipase was measured through the implementation of kits (Nanjing Jiancheng Biotechnology Co., Ltd., Nanjing, China).

### 2.10. Intestinal Antioxidant Activity

The intestinal samples were retrieved from a −80 °C ultra-low temperature freezer and thawed at 4 °C. After weighing, the samples were homogenized with normal saline at a ratio of 1:9 (g/mL). The homogenate was then centrifuged at 3000 r/min for 10 min at 4 °C. Subsequently, 500 µL of the supernatant was collected for analysis. Malondialdehyde (MDA) content was quantified using the thiobarbituric acid (TBA) method [49], whereby MDA forms a red adduct measured at 532 nm. Total antioxidant capacity (T-AOC) was assessed by a ferrous ion reduction method [50] and expressed relative to an FeSO_4_ standard. Catalase (CAT) activity was determined according to the method of Moretti et al. [51]. All assays, including MDA (nmol mg^−1^ protein), T-AOC (nmol mg^−1^ protein), and CAT activity (U mg^−1^ protein), were performed using commercial kits (Nanjing Jiancheng Bioengineering Institute, Nanjing, China) in accordance with the manufacturer’s instructions.

### 2.11. Intestine Histology

The foregut samples were retrieved from 70% ethanol. Subsequent processing involved washing, dehydration in a graded ethanol series, clearing in xylene, and embedding in paraffin wax. The embedded blocks were sectioned at 5 μm thickness and stained with hematoxylin and eosin (H&E) for histological analysis [52]. Morphometric measurements of villus height, crypt depth, and muscularis thickness were performed.

### 2.12. Intestinal Microbial Community

Samples were shipped to Shanghai Majorbio Bio-pharm Technology Co., Ltd. (Shanghai, China) for DNA extraction and Illumina MiSeq sequencing. Genomic DNA was extracted from 50 mg of thawed intestinal tissue using SLX-Mlus buffer according to the manufacturer’s protocol. Briefly, tissue was homogenized in 725 μL of ice-cold SLX-Mlus buffer for 5 min and centrifuged at 10,000 r/min for 5 min, and the supernatant was collected. DNA quality was assessed by 1% agarose gel electrophoresis and spectrophotometry. The 16S rRNA gene was amplified and sequenced on the Illumina MiSeq PE300 platform (San Diego, CA, USA). The raw 16S rRNA sequencing reads have been deposited in the NCBI Sequence Read Archive (SRA) database under BioSample accession numbers SAMN54928432–SAMN54928446.

### 2.13. Statistical Analysis

The data are presented as the mean ± standard error of the mean (SEM). The statistical analysis was carried out using SPSS 22.0 software. For general growth, serum, intestinal enzyme and antioxidant indices, one-way ANOVA was followed by Duncan’s multiple comparison test so that differences among groups were assessed. For the analysis of differentially abundant intestinal microbial taxa across the five treatment groups, one-way ANOVA was performed followed by Conover–Iman post hoc test to rigorously evaluate the intergroup variations in microbial relative abundance. Statistically significant differences were considered to have been found at *p* < 0.05.

## 3. Results

### 3.1. Nutritional Enhancement of Beet Pulp Through Mixed-Strain Solid-State Fermentation

Orthogonal experiment results indicated that treatment 3 (*Lactiplantibacillus plantarum*:*Saccharomycopsis fibuligera*:*Bacillus subtilis* = 1:3:3) achieved the highest comprehensive evaluation score (D = 15.64%), along with the highest crude protein (CP) increase rate (21.95%) and neutral detergent fiber (NDF) degradation rate (9.33%) (Table 3). Furthermore, range analysis revealed that the influence of factors on the comprehensive effect followed the order: B > C > A, with R values of 2.75, 1.82, and 0.84, respectively.

Subsequent chemical analysis confirmed that solid-state fermentation with the mixed microbial consortium significantly improved the nutritional profile of the beet pulp. The CP content increased from 7.88% to 9.61% (a 21.95% increase, *p* < 0.05), and the crude ash content was also significantly elevated (*p* < 0.05). In contrast, the crude fiber (CF) content decreased from 19.24% to 16.21% (a 15.75% reduction, *p* < 0.05), and the NDF content decreased from 46.47% to 42.13% (a 9.33% reduction, *p* < 0.05). Furthermore, the fermentation process promoted the production of organic acids, resulting in substantial accumulation of acetic, propionic, butyric, and lactic acids in the final product (Table 4).

The fermentation also significantly increased the amino acid content of the beet pulp (Table 5). Specifically, the total essential amino acids (ΣEAA) increased from 2.44% to 2.99% (a 22.54% increase, *p* < 0.05), and total amino acids (ΣTAA) increased from 5.24% to 6.20% (an 18.32% increase, *p* < 0.05). Levels of the individual essential amino acids, including leucine (Leu), isoleucine (Ile), and lysine (Lys), were all significantly elevated (*p* < 0.05).

In summary, mixed-strain fermentation markedly enhanced the nutritive value of beet pulp. Treatment 3 (microbial ratio of 1:3:3) yielded the optimal comprehensive D-value. Under this condition, a 21.95% increase in CP, alongside degradation rates of 15.75% for CF and 9.33% for NDF, was achieved. Concurrently, ΣEAA and ΣTAA were increased by 22.54% and 18.32%, respectively, indicating a substantial improvement in the protein quality and functional properties of the substrate. Based on these results, the optimal combination A1B3C3—comprising 2 mL of *L. plantarum*, 6 mL of *S. fibuligera*, and 6 mL of *B. subtilis*—was selected for feed formulation. The fermented beet pulp (FBP) was used to replace a portion of soybean meal in the experimental diets, which were prepared as outlined in Table 2. Proximate composition analysis confirmed that all diets had similar contents of crude protein, crude lipid, and crude ash (Table 6). Subsequently, a feeding trial was conducted with yellow catfish (*Pelteobagrus fulvidraco*) to evaluate the impact of FBP incorporation on growth performance, muscle quality, and serum biochemical parameters.

### 3.2. Growth Performance

The survival rate of yellow catfish was not significantly influenced by dietary supplementation with mixed solid-state fermented sugar beet pulp (FBP) (Table 7). Final body weight, weight gain rate (WGR), and specific growth rate (SGR) increased significantly (*p* < 0.05) with increasing FBP inclusion levels. Fish fed the RM9 diet demonstrated the best growth performance; however, the growth indices in the RM12 group were not significantly different from those in the RM9 group (*p* > 0.05). Furthermore, no significant differences were observed among dietary treatments for the condition factor (CF), viscerosomatic index (VSI), hepatosomatic index (HSI), or feed conversion ratio (FCR) (*p* > 0.05, Table 7).

### 3.3. Muscle Texture Profile

**Figure 1:**
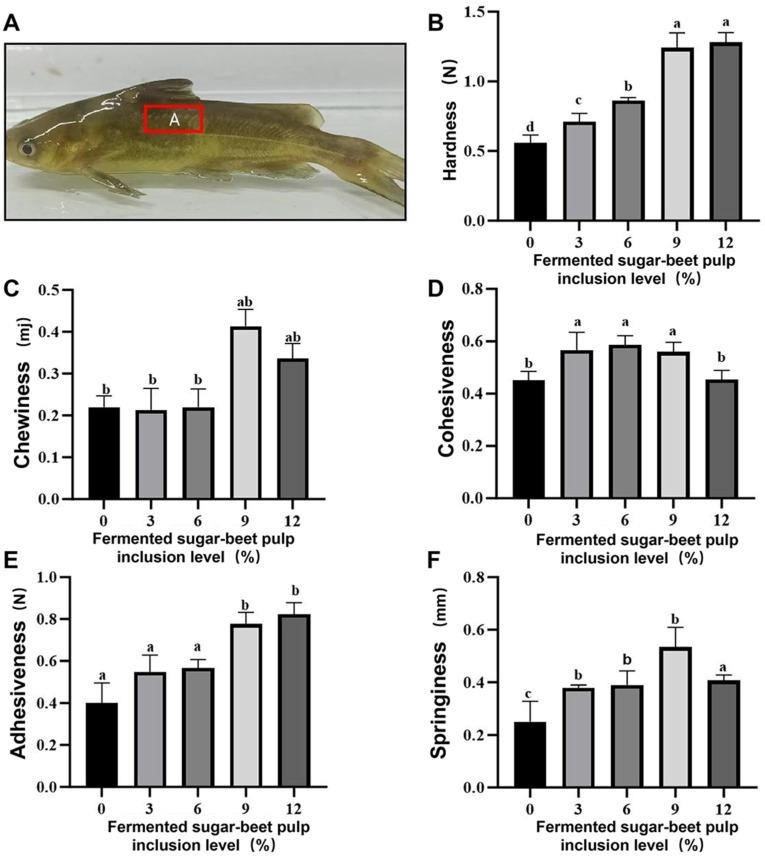
Effects of dietary fermented sugar beet pulp (FBP) inclusion levels on muscle texture parameters of juvenile yellow catfish (*Pelteobagrus fulvidraco*) (**A**) Schematic diagram of muscle sampling location; (**B**) Hardness, (**C**) Cohesiveness, (**D**) Elasticity, (**E**) Gumminess, (**F**) Chewiness of the dorsal muscle in different dietary groups. Muscle samples were collected from the dorsal musculature above the lateral line and cut into uniform blocks (1.00 cm × 1.00 cm × 0.50 cm) for texture profile analysis (TPA). Experimental groups: 0% (control, no FBP), 3% (RM3), 6% (RM6), 9% (RM9) and 12% (RM12) FBP replacing soybean meal, with 3 biological replicates per group. TPA was conducted with the instrument set at pre-test speed 2 mm/s, test speed 2 mm/s, post-test speed 2 mm/s and 20% deformation. Data are presented as mean ± standard error of the mean (SEM). Different lowercase letters in the upper right corner of the values indicate significant differences (*p* < 0.05) among different treatments for the same index. Values without such notations or with the same letters indicate no significant differences (*p* > 0.05).

Muscle texture parameters, including hardness, cohesiveness, gumminess, springiness, and chewiness, generally exhibited an increasing trend with higher dietary inclusion levels of mixed solid-state fermented sugar beet pulp (FBP), though the responses varied among specific parameters (Figure 1). Hardness was significantly elevated (*p* < 0.05) in fish fed the RM9 and RM12 diets compared to all other groups (Figure 1B). For cohesiveness, the RM3, RM6, and RM9 groups showed significantly higher values (*p* < 0.05) relative to the control (Figure 1D). The degree of springiness in the RM9 group was significantly greater than that observed in all other treatment groups (*p* < 0.05) (Figure 1F). The gumminess recorded was highest in the RM12 group (*p* < 0.05) (Figure 1E), while chewiness was greatest in the RM9 group (*p* < 0.05) (Figure 1C).

### 3.4. Serum Biochemical Indices

Dietary supplementation with fermented sugar beet pulp (FBP) significantly influenced several serum biochemical parameters in yellow catfish (Table 8). No statistically significant differences were observed in the levels of serum total protein (TP), albumin (ALB), or urea nitrogen (BUN) among the dietary groups (*p* > 0.05). Conversely, serum glucose (GLU) levels were significantly elevated in the RM12 group in comparison to the control group (*p* < 0.05). Regarding hepatic health markers, we found that serum aspartate aminotransferase (AST) activity was significantly lower in all groups receiving FBP compared to the control group (*p* < 0.05), whereas alanine aminotransferase (ALT) activity showed no notable change (*p* > 0.05). FBP supplementation also appeared to improve lipid metabolism. Both serum total cholesterol (T-CHO) and triglyceride (TG) levels decreased as more FBP was included in the diet, with the RM9 and RM12 groups showing significantly lower values than the control (*p* < 0.05)—the lowest values were recorded in RM12 group. In terms of antioxidant status, serum catalase (CAT) activity increased progressively with higher FBP inclusion, peaking in the RM12 group, where it was significantly elevated compared to the control (*p* < 0.05). At the same time, serum malondialdehyde (MDA) content—an indicator of lipid peroxidation—was significantly lower in the RM9 and RM12 groups (*p* < 0.05), suggesting that oxidative damage was reduced.

### 3.5. Intestinal Digestive Enzymes, Antioxidant Activity, and Microbial Community

Intestinal lipase and trypsin activities were significantly higher in RM6 and RM9 groups compared to the control (*p* < 0.05), with the highest activity observed in the RM9 group, while intestinal amylase activity increased significantly with dietary fermented sugar beet pulp inclusion levels when the addition exceeded RM3 (*p* < 0.05), reaching the maximum value in the RM12 group (Table 9).

Dietary supplementation with solid-state fermented sugar beet pulp using a mixed consortium significantly influenced hepatic antioxidant capacity in yellow catfish (*Pelteobagrus fulvidraco*) (Table 10). Total antioxidant capacity (T-AOC) and catalase (CAT) activities were significantly higher in yellow catfish fed the RM9 and RM12 diets than in the control (*p* < 0.05), with the highest values observed in the RM12 group. In contrast, malondialdehyde (MDA) content was significantly reduced (*p* < 0.05), and the lowest MDA content was observed in the RM9 group.

**Figure 2:**
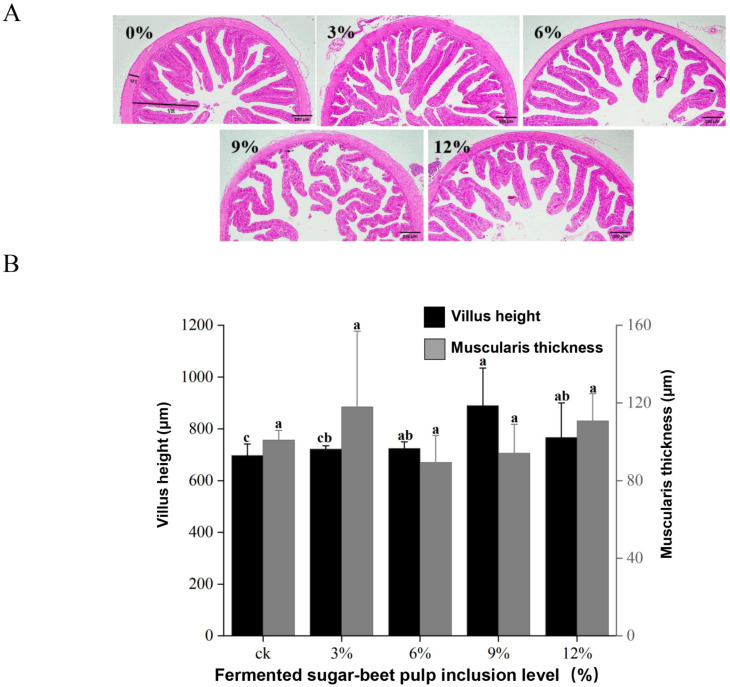
Intestinal histomorphology of juvenile yellow catfish (*Pelteobagrus fulvidraco*) fed diets with different fermented sugar beet pulp (FBP) inclusion levels. (**A**) Hematoxylin and eosin (H&E) stained foregut tissue sections (scale bar consistent across all groups); (**B**) Quantitative analysis of intestinal villus height and muscularis thickness. Experimental groups: CK (0% FBP, control), F1 (3% FBP), F2 (6% FBP), F3 (9% FBP), F4 (12% FBP), with 3 biological replicates per group. VH = villus height, MT = muscularis thickness. Data are presented as mean ± standard error of the mean (SEM). Different lowercase letters above the bars indicate significant differences among groups (one-way ANOVA followed by Duncan’s multiple comparison test, *p* < 0.05).

Histological observation of intestinal morphology revealed progressive development of villi in RM3, RM6, RM9, and RM12 groups, characterized by increased extension, enhanced branching, and elongated structure (Figure 2A). When compared with the control group, villus length was significantly elevated in RM6, RM9, and RM12 groups (*p* < 0.05), reaching maximal values in the RM9 group (Figure 2B). By contrast, the foregut muscular layer thickness was not significantly influenced by dietary treatments (*p* > 0.05) (Figure 2B).

**Figure 3:**
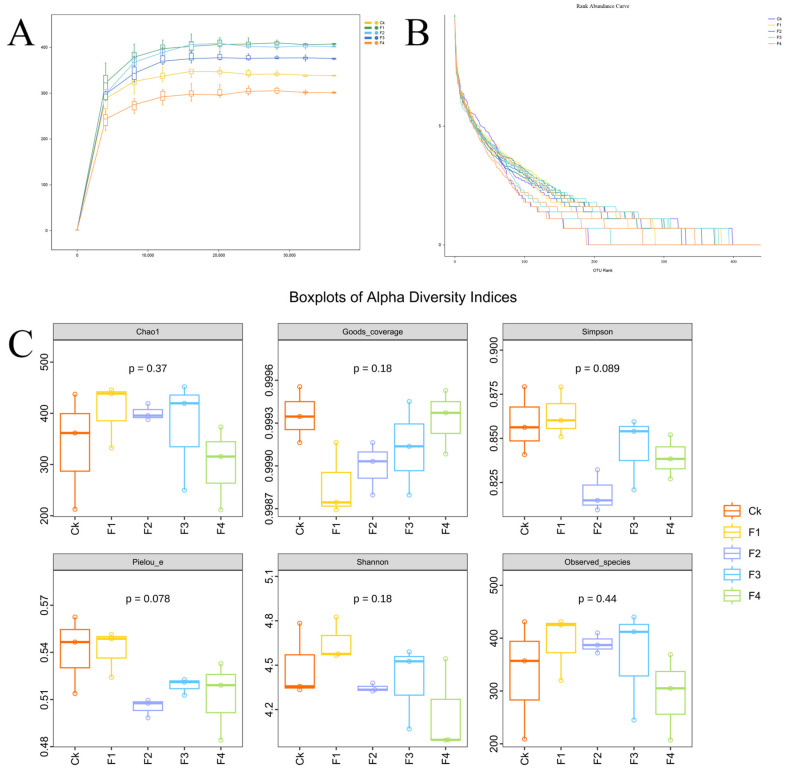
Alpha diversity analysis of intestinal microbiota in juvenile yellow catfish (*Pelteobagrus fulvidraco*) fed diets with different fermented sugar beet pulp (FBP) inclusion levels (**A**) Species accumulation curve; (**B**) Rank abundance curve; (**C**) Boxplots of alpha diversity indices (Chao1, Good’s coverage, Simpson, Pielou_e, Shannon, Observed_species). Experimental groups: CK (0% FBP, control), F1 (3% FBP), F2 (6% FBP), F3 (9% FBP), F4 (12% FBP), with 3 biological replicates per group. Good’s coverage values close to 1 indicate sufficient sequencing depth. *p*-values above the boxplots indicate the significance of intergroup differences (one-way ANOVA, *p* < 0.05 considered significant).

Here, alpha diversity was comprehensively assessed through indices of species richness (Chao1 and Observed species), coverage (Good’s coverage), diversity (Shannon and Simpson indices), and evenness (*Pielou_e*) within a single homogeneous habitat. The results revealed that the total species number on the accumulation curve was slightly lower in the Ck group than in the experimental groups (F1, F2, and F3) (Figure 3A), while species abundance curves for all groups displayed a steep declining trend (Figure 3B). However, statistical analysis indicated no significant differences between the control and experimental groups in any of the assessed indices, including Chao1, Observed species, Shannon, Simpson, Good’s coverage (which was close to 1 for all groups), or *Pielou_e* (Figure 3C).

**Figure 4:**
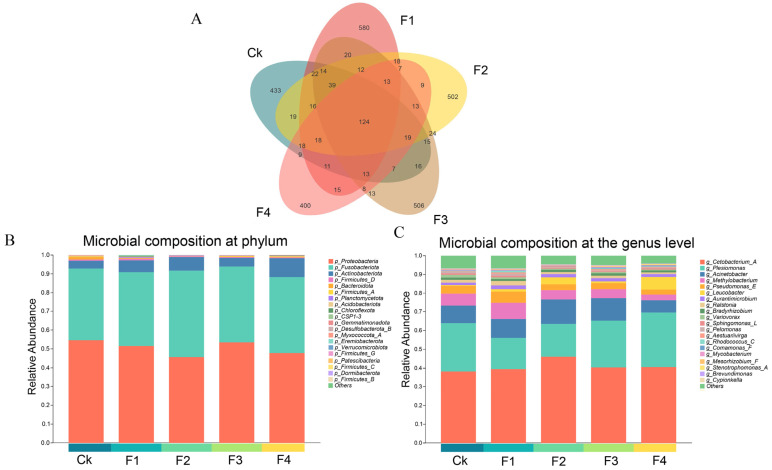
Intestinal microbial community composition of juvenile yellow catfish (*Pelteobagrus fulvidraco*) fed diets with different fermented sugar beet pulp (FBP) inclusion levels. (**A**) Venn diagram showing the number of unique and shared operational taxonomic units (OTUs) among groups; (**B**) Relative abundance of intestinal microbiota at the phylum level (only dominant phyla with relative abundance >0.01 are shown, others are pooled as “Others”); (**C**) Relative abundance of intestinal microbiota at the genus level (only dominant genera with relative abundance >0.01 are shown, others are pooled as “Others”). Experimental groups: CK (0% FBP, control), F1 (3% FBP), F2 (6% FBP), F3 (9% FBP), F4 (12% FBP), with 3 biological replicates per group. Microbial DNA was extracted from foregut tissue, and 16S rRNA gene sequencing (V4-V5 region) was performed on the Illumina MiSeq PE300 platform.

Microbial community analysis revealed distinct patterns across different taxonomic levels among experimental groups (Figure 4). At the OTU level, a total of 124 OTUs were shared across all experimental groups, while the number of unique OTUs varied substantially among groups (Figure 4A). Group F1 exhibited the highest number of unique OTUs (580), followed by group F3 (506) and group F2 (502), whereas groups Ck and F4 possessed 433 and 400 unique OTUs, respectively. Moving to broader taxonomic classification, microbial communities at the phylum level were predominantly composed of *Proteobacteria*, *Fusobacteria*, *Actinobacteria*, and *Firmicutes* (Figure 4B and Figure S1A). At a finer taxonomic resolution, the dominant bacterial genera were relatively consistent across all groups, primarily comprising *Cetobacterium*, *Plesiomonas*, *Acinetobacter*, and *Methylobacterium* (Figure 4C). To identify taxa with significant differences in abundance across the five dietary groups, one-way ANOVA followed by the Conover–Iman post hoc test was conducted. The ANOVA results revealed significant variations among groups at the phylum level for *Actinobacteria* (*p* = 0.01134), *Fusobacteriota* (*p* = 0.030423) and Proteobacteria (*p* = 0.016). At the genus level, significant differences were detected for *Ralstonia* (*p* = 0.006), *Leucobacter* (*p* = 0.003), *Pseudomonas* (*p* = 0.024), *Acinetobacter* (*p* = 0.035), *Plesiomonas* (*p* = 0.016), and *Cetobacterium* (*p* = 0.031). Although the genus *Cetobacterium* reached its highest relative abundance in the F2 group (46.09%), which was 7.85 percentage points higher than in the control group (38.24%) (Figure 4C and Figure S1B), the post hoc test confirmed that this numerical increase was not statistically significant (*p* > 0.05) (Figure S2). In contrast, post hoc analysis further revealed that *Leucobacter* was most significantly enriched in the F4 (12% FBP) group, with its abundance being significantly higher than in all other groups (Figure S2).

## 4. Discussion

This study demonstrates that solid-state fermentation using a specific microbial consortium can effectively convert sugar beet pulp into an ingredient with enhanced nutritional value and functional activity. The orthogonal optimization results provided important insights into the fermentation process. The inoculation ratio of *L. plantarum*, *S. fibuligera*, and *B. subtilis* significantly affected the fermentation outcome. The optimal volume ratio of 1:3:3 yielded the highest comprehensive evaluation D-value (15.67%), primarily driven by a 21.95% increase in crude protein content and a 9.33% reduction in neutral detergent fiber. These nutritional improvements can be explained by distinct microbial mechanisms. This CP enrichment is likely attributable to microbial biomass (single-cell protein) synthesis utilizing the carbon and nitrogen sources present in SBP, a common phenomenon in microbial fermentation of plant substrates [53]. Meanwhile, the degradation of fibrous components, including crude fiber and neutral detergent fiber, can be attributed to the synergistic action of extracellular enzymes—particularly cellulases and hemicellulases secreted by *B. subtilis* and *S. fibuligera*. These enzymes break down complex structural polysaccharides into simpler, more digestible molecules [54].

Beyond macronutrient improvements, solid-state fermentation also significantly enhanced the protein quality of sugar beet pulp. We observed notable increases in both total amino acids (TAA) and essential amino acids (EAA), with particularly prominent rises in leucine, lysine, isoleucine, and arginine. The biological significance of these amino acids for aquaculture species is well established: leucine acts as a key regulator of muscle protein synthesis; lysine is often the first limiting amino acid in plant-based diets; arginine enhances immunity and serves as a precursor for nitric oxide; while isoleucine is involved in energy metabolism [55]. The improved amino acid profile likely results from a dual mechanism: first, the hydrolysis of native SBP proteins into peptides and free amino acids by microbial proteases; and second, the synthesis and subsequent release of amino acids by proliferating microbial cells themselves [56].

Furthermore, the solid-state fermentation process led to a substantial accumulation of organic acids, including lactic, propionic, and butyric acids, which increased the total volatile fatty acid content to 5.74 × 10^3^ mg/kg. This quantitative increase in organic acids directly imparts significant functional properties to the fermented beet pulp (FBP), as these metabolites are not merely fermentation by-products but also active functional compounds. Lactic acid, primarily produced by *L. plantarum*, serves as a feed acidifier that effectively inhibits enteropathogens [57]. Meanwhile, propionic and butyric acids act as important energy sources for intestinal epithelial cells and play key roles in maintaining mucosal integrity and barrier function in fish [58]. Together, these comprehensive enhancements in the nutrition and functionality of FBP establish the biochemical foundation that directly promotes growth performance and improves health status in fish.

The nutritional improvements in FBP translated directly into enhanced zootechnical performance in yellow catfish. Dietary inclusion of FBP significantly improved final body weight, weight gain rate (WGR), and specific growth rate (SGR) in a generally dose-dependent manner up to 9% inclusion (RM9 diet). The growth-promoting effect can be attributed to the composite action of multiple bioactive compounds in FBP. Firstly, organic acids likely improved the palatability of the diet and enhanced gut health by lowering luminal pH, modulating the microbiota, and increasing the activity of endogenous digestive enzymes, thereby improving nutrient digestibility and absorption [59]. Secondly, the pre-digestive action of microbial enzymes in FBP may have increased the bioavailability of nutrients from the overall diet matrix [60]. Thirdly, the high-quality microbial protein and readily available peptides in FBP provided excellent amino acid sources for protein accretion. However, our findings indicate that growth performance reached a plateau at the 12% inclusion level (RM12), with no statistically significant advantage over the 9% level (RM9), suggesting a potential optimal inclusion threshold. Beyond this point, even though fermentation partially degrades the fiber content in FBP, its rising proportion may start to dilute dietary energy density or mildly hinder nutrient utilization—a practical consideration commonly observed when incorporating fibrous materials into aquafeeds [61]. It is noteworthy that, across all inclusion levels tested (3–12%), FBP did not adversely affect survival, feed conversion ratio (FCR), or somatic indices (condition factor, viscerosomatic index, or hepatosomatic index). These results affirm the safety and palatability of FBP and suggest that it does not trigger metabolic or physiological stress that could interfere with energy allocation or organ development.

In addition to promoting growth, dietary inclusion of FBP significantly enhanced fillet quality. Muscle texture serves as a vital quality indicator in aquatic products, directly influencing tenderness, springiness, and sensory properties [62]. Hardness measures the muscle’s resistance to deformation and indicates structural integrity, while cohesiveness reflects the binding strength among muscle fibers. Springiness and resilience, in turn, capture the ability of muscle to recover its original shape after force is applied—together determining whether the eating experience is crisp and tender or soft and mushy [63]. While environmental and farming practices also affect muscle texture, diet has been recognized as a primary modulating factor [64]. Consistent with this principle, fermented beet pulp appears to improve fillet quality by enhancing muscle springiness, hardness, textural stability, and overall firmness. These improvements may be attributed to bioactive compounds produced during fermentation—including proteases, peptides, and organic acids—which could facilitate protein cross-linking or modify interactions between muscle fibers.

The benefits in growth and fillet quality were further supported by broader systemic and intestinal health improvements associated with FBP. Notably, we observed a significant decrease in serum aspartate aminotransferase (AST) activity across all FBP-fed groups. Since AST is a reliable marker of hepatocellular integrity, its reduction suggests a hepatoprotective effect, potentially resulting from lowered metabolic burden or the antioxidant activity of FBP [65,66]. Moreover, FBP supplementation favorably influenced lipid metabolism, significantly reducing serum total cholesterol (T-CHO) and triglyceride (TG)—especially in the higher inclusion groups (RM9 and RM12). In yellow catfish, these lipid parameters reflect metabolic regulation capacity, and their decline indicates improved utilization of dietary fats by tissues [67]. This lipid-lowering effect may stem from the elevated dietary fiber in FBP, which could promote bile acid excretion and thus lower serum lipids, or from fermentation-derived compounds that modulate hepatic lipogenesis [68]. In parallel, FBP boosted systemic antioxidant defenses. Our study showed that FBP supplementation significantly increased serum antioxidant capacity (measured as CAT activity) while reducing the oxidative damage marker MDA. These results point to clear antioxidant properties of fermented beet pulp, capable of strengthening antioxidant defenses and mitigating oxidative stress [69,70,71,72].

The health-promoting effects of FBP were especially evident at the intestinal level, which plays a central role in nutrient utilization. Digestive enzyme activity is a key functional gauge of feed efficiency. Here, FBP supplementation markedly increased intestinal lipase, trypsin, and amylase activities in yellow catfish, with the strongest effects seen in RM9 and RM12 groups. This pattern closely tracked the improved growth, suggesting that FBP enhances digestive function, raises feed utilization efficiency, and thereby supports growth [73,74]. Complementing these functional gains, intestinal structure also improved. We found a significant increase in villus length in the RM6, RM9, and RM12 groups, peaking in RM9, indicating that FBP primarily boosts intestinal absorptive capacity. This structural improvement may be linked to small-molecule peptides released during fermentation, which prior studies confirm can stimulate brush-border enzyme activity and promote villus growth [75,76]. Together, these functional and structural improvements highlight that FBP promotes nutrient use and growth through complementary functional and structural mechanisms in the intestine.

During metabolism, excess free radicals can lead to oxidative damage [77]. To counteract this challenge, animals possess a defense system comprising antioxidant enzymes and reductive substances [78]. The T-AOC level reflects the overall antioxidant capacity of fish [79]. CAT is a key antioxidant enzyme that alleviates oxidative stress by breaking down hydrogen peroxide, thereby protecting intestinal tissues [70]. MDA, a lipid peroxidation product, generally varies inversely with antioxidant enzyme levels [80]. In our trial, RM9 and RM12 groups showed significantly higher T-AOC and CAT activity—with CAT peaking in RM12—while MDA content dropped to its lowest in RM9. This confirms that fermented beet pulp effectively reduced oxidative stress and strengthened antioxidant capacity in yellow catfish. Similar antioxidant benefits have been reported with fermented soybean meal in white shrimp [81], and fermented rapeseed meal in juvenile red sea bream [72]. The underlying mechanism may involve the intestinal absorption of antioxidant compounds produced during fermentation, thereby mitigating oxidative stress [27].

Another important mechanism by which FBP supports intestinal health is through modulation of the gut microbiota, a vital ecosystem for host health [82]. Our data indicate that FBP did not significantly alter alpha diversity or dominant phyla composition, implying a relatively gentle effect on overall microbial structure. However, one-way ANOVA revealed significant differences in the relative abundances of *Actinobacteriota*, *Fusobacteriota*, and *Proteobacteria* at the phylum level (*p* < 0.05). At the genus level, statistical analysis identified significant differences in the relative abundances of several key taxa among groups (*p* < 0.05). Post hoc analysis further confirmed that *Leucobacter* was most significantly enriched in the F4 (12% FBP) group, with its abundance being significantly higher than that in all other groups. This finding is particularly significant because *Leucobacter*—a core taxon identified in the intestines of various fish species—has been associated with potential probiotic functions, including carotenoid production and antioxidant activity [83]. Recent studies have also demonstrated that specific probiotic treatments can enhance the intestinal abundance of *Leucobacter* and improve host health outcomes [84]. Notably, *Cetobacterium*, a common anaerobic genus in fish intestines, plays a key role in producing vitamin B12 and participating in protein and carbohydrate metabolism, thereby contributing to host nutrition and intestinal health [85,86]. Although no statistically significant pairwise differences were observed for this genus among the treatment groups in the present study, the overall trend in its abundance warrants further attention in future research.

Taken together with the observed improvements in intestinal morphology, digestive function, and antioxidant status, these findings suggest that FBP modulates the gut microbiota in a taxon-specific manner—selectively influencing functional microbial groups involved in nutrient metabolism rather than inducing broad structural changes. This precise modulation may contribute to an optimized intestinal microenvironment and support host health.

## 5. Conclusions

In summary, solid-state fermentation with *L. plantarum*, *S. fibuligera*, and *B. subtilis* consortium (1:3:3 ratio) effectively valorizes sugar beet pulp into a high-value functional feed ingredient for yellow catfish (*Pelteobagrus fulvidraco*). Based on the comprehensive evaluation of growth performance, muscle quality, and health parameters, the optimal dietary inclusion level was determined to be 9%. At this inclusion level, fermented sugar beet pulp enhanced growth performance, improved muscle textural quality, supported hepatic and systemic health, and promoted intestinal function. From a practical application perspective, although fermented sugar beet pulp remains a low-protein ingredient compared to soybean meal and cannot directly replace high-protein ingredients such as soybean meal, this fermented product can be strategically utilized in combination with protein-rich ingredients or even animal-derived protein sources. Such complementary use can reduce the proportion of high-cost protein sources in aquafeeds while ensuring the nutritional requirements of aquatic animals are met, thereby decreasing feed costs. Overall, these findings provide a scientific basis for utilizing mixed-strain fermented sugar beet pulp as a sustainable alternative to partially replace soybean meal in aquafeeds.

## Figures and Tables

**Table 1:** Orthogonal experimental design of the influence of different strain ratios on cp growth rate, ndf degradation rate and comprehensive effect D.

Treatment	A	B	C	A:B:C
1	2 mL	2 mL	2 mL	1:1:1
2	2 mL	4 mL	4 mL	1:2:2
3	2 mL	6 mL	6 mL	1:3:3
4	4 mL	2 mL	6 mL	2:1:3
5	4 mL	4 mL	2 mL	2:2:1
6	4 mL	6 mL	4 mL	2:3:2
7	6 mL	2 mL	4 mL	3:1:2
8	6 mL	4 mL	6 mL	3:2:3
9	6 mL	6 mL	2 mL	3:3:1

**Table 2:** Formulation table of the yellow catfish culture experiment (%).

Ingredients (%)	Control	RM3	RM6	RM9	RM12
fish meal	30	30	30	30	30
soybean meal	31	25.7	20.4	15.1	9.8
corn gluten meal	5	5	5	5	5
soy protein isolate	6.5	8.8	11.1	13.4	15.7
choline chloride	0.4	0.4	0.4	0.4	0.4
sodium L-ascorbyl-2-phosphate	0.2	0.2	0.2	0.2	0.2
lutein	0.4	0.4	0.4	0.4	0.4
fish oil	5	5	5	5	5
vitamin premix ^a^	0.5	0.5	0.5	0.5	0.5
mineral premix ^b^	0.5	0.5	0.5	0.5	0.5
calcium dihydrogen phosphate	2.5	2.5	2.5	2.5	2.5
wheat flour	18	18	18	18	18
fermented sugar beet pulp powder	0	3	6	9	12
Total	100	100	100	100	100

1. vitamin premix ^a^ (mg or IU per kg diet): retinyl acetate, 4500 IU; cholecalciferol, 1000 IU; all-rac-a-tocopheryl acetate, 200; menadione nicotinamide bisulfite, 40; ascorbyl-2-polyphosphate, 25; thiamine hydrochloride, 28; riboflavin, 80; pyridoxine hydrochloride, 40; D-calcium pantothenate, 80; folic acid, 4; biotin, 0.2; cyanocobalamin, 0.01; inositol, 80; choline chloride, 500. 2. mineral premix ^b^ (mg per kg diet): NaCl, 1000; FeSO_4_·7H_2_O, 180; ZnSO_4_·7H_2_O, 70; MgSO_4_·7H_2_O, 50; MnSO_4_·H_2_O, 28; CuSO_4_·5H_2_O, 3.9; CoCl_2_, 0.89; KI, 0.8.

**Table 3:** The influence of different strain ratios on cp growth rate, ndf degradation rate and comprehensive effect.

Treatment	A	B	C	CP (%)	NDF (%)	D (%)
1	1	1	1	12.89	6.88	9.85
2	1	2	2	15.58	7.95	11.76
3	1	3	3	21.95	9.33	15.64
4	2	1	3	15.58	8.5	12.04
5	2	2	1	15.34	7.21	11.28
6	2	3	2	18.43	8.33	13.38
7	3	1	2	13.24	7.10	10.17
8	3	2	3	15.57	9.4	12.49
9	3	3	1	16.54	7.74	12.14
k1	12.43	10.7	11.1	-	-	-
k2	12.23	11.84	11.77	-	-	-
K3	11.6	13.72	12.93	-	-	-
range (R)	0.83	3.02	2.29	-	-	-
Optimal level	A_1_B_3_C_3_	-	-	-	-	-
ranking	B > C > A	-	-	-	-	-

**Table 4:** Effect of Fermentation Treatment on the Component Content of Beet Pulp.

Items	Unfermented Sample	Fermented Sample
crude protein (%)	7.88 ± 0.22 ^b^	9.61 ± 0.13 ^a^
crude fiber (%)	19.24 ± 0.27 ^b^	16.21 ± 0.31 ^a^
neutral detergent fiber (%)	46.47 ± 0.48 ^b^	42.13 ± 0.33 ^a^
crude ash (%)	4.55 ± 0.07 ^b^	5.02 ± 0.08 ^a^
acetic acid (mg/kg)	-	5.68 × 10^3^ ± 10
propionic acid (mg/kg)	-	15.63 ± 0.65
butyric acid (mg/kg)	-	7.6 ± 0.2
lactic acid (mg/kg)	-	9.06 × 10^3^ ± 40
total volatile fatty acids (mg/kg)	-	5.74 × 10^3^ ± 10

Note: Different lowercase letters in the upper right corner of the values indicate significant differences (*p* < 0.05) among different treatments for the same index. Values without such notations or with the same letters indicate no significant differences (*p* > 0.05).

**Table 5:** The influence of fermentation treatment on the amino acid composition and essential amino acid content of sugar beet pulp (g/100 g).

Items	Unfermented Sample	Fermented Sample
Asp	0.50 ± 0.03	0.55 ± 0.02
Thr *	0.33 ± 0.03	0.37 ± 0.01
Ser	0.33 ± 0.03	0.36 ± 0.01
Glu	0.70 ± 0.05	0.84 ± 0.08
Gly	0.30 ± 0.03	0.34 ± 0.01
Ala	0.32 ± 0.03	0.36 ± 0.01
Cys	0.04 ± 0.01	0.05 ± 0.10
Val *	0.42 ± 0.03	0.48 ± 0.02
Met *	0.06 ± 0.02	0.08 ± 0.05
Ile *	0.25 ± 0.01 ^a^	0.29 ± 0.02 ^b^
Leu *	0.38 ± 0.03 ^a^	0.45 ± 0.03 ^b^
Tyr	0.27 ± 0.03	0.30 ± 0.03
Phe *	0.26 ± 0.02 ^a^	0.30 ± 0.01 ^b^
Lys *	0.34 ± 0.06	0.49 ± 0.11
His *	0.20 ± 0.02 ^a^	0.24 ± 0.01 ^b^
Arg *	0.21 ± 0.04 ^a^	0.31 ± 0.04 ^b^
pro	0.34 ± 0.05	0.39 ± 0.07
∑TAA	5.24 ± 0.48 ^a^	6.20 ± 0.33 ^b^
∑EAA	2.44 ± 0.20 ^a^	2.99 ± 0.19 ^b^

Note: * Represents essential amino acids, EAA represents the total essential amino acids for fish, and TAA represents the total amino acids. Different lowercase letters in the upper right corner of the values indicate significant differences (*p* < 0.05) among different treatments for the same index. Values without such notations or with the same letters indicate no significant differences (*p* > 0.05).

**Table 6:** Contents of some nutritional components in the feed formula (measured values, %).

Ingredients	Control	RM3	RM6	RM9	RM12
crude protein (%)	41.65	41.47	41.38	41.29	41.24
crude fat (%)	8.75	8.71	8.68	8.72	8.69
crude ash (%)	10.19	10.24	10.17	10.11	10.21

**Table 7:** Effect of Different Proportions of Fermented Beet Pulp on Growth Performance and Feed Conversion Efficiency of Yellow Catfish.

Items	Control	RM3	RM6	RM9	RM12
SR (%)	100	100	100	100	100
initial weight (g)	3.56 ± 0.03	3.57 ± 0.03	3.55 ± 0.02	3.56 ± 0.04	3.56 ± 0.05
final weight (g)	10.26 ± 0.94 ^c^	11.94 ± 0.32 ^ab^	12.95 ± 0.39 ^ab^	13.68 ± 0.46 ^a^	13.85 ± 0.89 ^a^
WGR (%)	188 ± 26.51 ^c^	236 ± 8.72 ^b^	262.67 ± 11.06 ^ab^	284.33 ± 13.01 ^a^	285 ± 17.35 ^a^
SGR (%)	1.76 ± 0.16 ^c^	2.02 ± 0.04 ^b^	2.15 ± 0.06 ^b^	2.25 ± 0.06 ^a^	2.26 ± 0.1 ^a^
CT (g/cm^3^)	1.39 ± 0.05	1.42 ± 0.08	1.49 ± 0.06	1.54 ± 0.09	1.52 ± 0.07
VSI (%)	7.48 ± 0.22	7.7 ± 0.11	7.38 ± 0.34	7.31 ± 0.34	7.55 ± 0.41
HIS (%)	1.36 ± 0.04	1.47 ± 0.06	1.49 ± 0.07	1.46 ± 0.08	1.51 ± 0.06
FCR	1.46 ± 0.05	1.40 ± 0.02	1.41 ± 0.01	1.43 ± 0.07	1.45 ± 0.03

Note: Different lowercase letters in the upper right corner of the values indicate significant differences (*p* < 0.05) among different treatments for the same index. Values without such notations or with the same letters indicate no significant differences (*p* > 0.05).

**Table 8:** The influence of different addition amounts of fermented sugar beet pulp on serum biochemical indexes.

Items	Control	RM3	RM6	RM9	RM12
TP (g/L)	40.39 ± 4.24	39.44 ± 2.86	37.07 ± 1.81	36.82 ± 3.91	35.23 ± 3.70
ALB (g/L)	12.15 ± 0.95	11.18 ± 1.15	10.63 ± 0.62	10.35 ± 0.97	9.79 ± 0.3
BUN (mmol/L)	2.43 ± 0.25	2.23 ± 0.15	2.27 ± 0.42	2.33 ± 0.21	2.13 ± 0.13
GLU (mmol/L)	6.52 ± 0.79 ^b^	6.11 ± 0.85 ^b^	5.88 ± 0.52 ^b^	6.61 ± 0.78 ^b^	7.96 ± 0.49 ^a^
AST (mmol/L)	542.33 ± 47.01 ^b^	461.00 ± 27.06 ^a^	427.67 ± 44.41 ^a^	415.00 ± 41.90 ^a^	437.00 ± 26.66 ^a^
ALT (mmol/L)	34.33 ± 3.06	30.67 ± 3.79	29.00 ± 2.00	32.67 ± 3.21	30.00 ± 3.61
T-CHO (mmol/L)	6.74 ± 0.49 ^a^	6.55 ± 0.38 ^a^	6.43 ± 0.36 ^a^	5.52 ± 0.48 ^b^	5.21 ± 0.24 ^b^
TG (mmol/L)	6.19 ± 0.37 ^a^	5.57 ± 0.40 ^b^	5.69 ± 0.28 ^ab^	5.09 ± 0.25 ^bc^	4.84 ± 0.30 ^c^
CAT (U/mL)	4.88 ± 0.66 ^a^	5.46 ± 0.33 ^ab^	5.62 ± 0.70 ^ab^	6.02 ± 0.54 ^ab^	6.77 ± 0.32 ^b^
MAD (nmol/L)	6.02 ± 0.43 ^a^	5.39 ± 1.27 ^ab^	3.53 ± 1.01 ^ab^	3.41 ± 0.61 ^b^	4.00 ± 0.69 ^b^

Note: Different lowercase letters in the upper right corner of the values indicate significant differences (*p* < 0.05) among different treatments for the same index. Values without such notations or with the same letters indicate no significant differences (*p* > 0.05).

**Table 9:** Effects of different amounts of fermented beet meal on intestinal digestive enzymes of *Pelteobagrus fulvidraco*.

Ingredients	Control	RM3	RM6	RM9	RM12
Intestinal lipase (U/gprot)	48.70 ± 3.59 ^c^	54.80 ± 4.25 ^bc^	62.9 ± 8.53 ^ab^	69.98 ± 3.72 ^a^	66.22 ± 3.37 ^a^
Intestinal trypsin (U/mgprot)	209.78 ± 12.02 ^c^	246.22 ± 8.15 ^b^	257.78 ± 13.68 ^ab^	271.11 ± 9.37 ^a^	268.44 ± 12.60 ^a^
Intestinal amylase (U/mgprot)	0.71 ± 0.06 ^c^	0.87 ± 0.13 ^c^	1.05 ± 0.12 ^b^	1.17 ± 0.10 ^ab^	1.28 ± 0.04 ^a^

Note: Different lowercase letters in the upper right corner of the values indicate significant differences (*p* < 0.05) among different treatments for the same index. Values without such notations or with the same letters indicate no significant differences (*p* > 0.05).

**Table 10:** Effects of different fermented beet meal additions on intestinal antioxidants of *Pelteobagrus fulvidraco*.

Ingredients	Control	RM3	RM6	RM9	RM12
T-AOC (nmol/mgprot)	0.43 ± 0.04 ^c^	0.48 ± 0.03 ^bc^	0.52 ± 0.06 ^abc^	0.55 ± 0.02 ^ab^	0.60 ± 0.05 ^a^
CAT (U/mgprot)	1.76 ± 0.09 ^c^	1.96 ± 0.18 ^bc^	2.25 ± 0.33 ^bc^	2.42 ± 0.10 ^b^	3.05 ± 0.26 ^a^
MDA (nmol/mgprot)	2.89 ± 0.14 ^a^	2.20 ± 0.26 ^b^	1.77 ± 0.09 ^b^	1.64 ± 0.39 ^b^	1.82 ± 0.19 ^b^

Note: Different lowercase letters in the upper right corner of the values indicate significant differences (*p* < 0.05) among different treatments for the same index. Values without such notations or with the same letters indicate no significant differences (*p* > 0.05).

## Data Availability

The raw 16S rRNA sequencing data of yellow catfish intestinal microbiota generated in this study have been deposited in the NCBI SRA database under BioSample accession numbers PRJNA1415281.

## Supplementary Materials

The following supporting information can be downloaded at: https://www.mdpi.com/article/10.3390/ani16060915/s1, Figure S1. Intestinal microbial community composition of juvenile yellow catfish (*Pelteobagrus fulvidraco*) at the phylum and genus levels following dietary fermented sugar beet pulp (FBP) supplementation. Figure S2. Differentially abundant intestinal microbial taxa in juvenile yellow catfish (*Pelteobagrus fulvidraco*) fed diets with different fermented sugar beet pulp (FBP) inclusion levels.

## References

[1] Ji Z., Lu X., Xue M., Fan Y., Tian J., Dong L., Zhu C., Wen H., Jiang M. (2023). The probiotic effects of host-associated Bacillus velezensis in diets for hybrid yellow catfish (*Pelteobagrus fulvidraco* ♀ × *Pelteobagrus vachelli* ♂). Anim. Nutr..

[2] Chen Q., Zhao H., Huang Y., Cao J., Wang G., Sun Y., Li Y. (2016). Effects of dietary arginine levels on growth performance, body composition, serum biochemical indices and resistance ability against ammonia-nitrogen stress in juvenile yellow catfish (*Pelteobagrus fulvidraco*). Anim. Nutr..

[3] Li X., Wang S., Zhang M., Jiang H., Qian Y., Wang R., Li M. (2023). Comprehensive analysis of metabolomics on flesh quality of yellow catfish *(Pelteobagrus fulvidraco*) fed plant-based protein diet. Front. Nutr..

[4] Chen Z., Fei S., Duan Y., Liu C., Liu H., Han D., Jin J., Yang Y., Zhu X., Xie S. (2022). Effects of dietary protein level on the growth, reproductive performance, and larval quality of female yellow catfish (*Pelteobagrus fulvidraco*) broodstock. Aquac. Rep..

[5] Zhou Q.-C., Zhao J., Li P., Wang H.-L., Wang L.-G. (2011). Evaluation of poultry by-product meal in commercial diets for juvenile cobia (*Rachycentron canadum*). Aquaculture.

[6] Hotchkiss A.T., Qi P., Liu L.S., Chau H.K., Cooke P.H., Nuñez A., White A.K., Fishman M.L. (2019). Sugar beet pulp fiber is a source of bioactive food and feed ingredients. Int. Sugar J..

[7] Wilkowska A., Berlowska J., Nowak A., Motyl I., Antczak-Chrobot A., Wojtczak M., Kunicka-Styczyńska A., Binczarski M., Dziugan P. (2020). Combined Yeast Cultivation and Pectin Hydrolysis as an Effective Method of Producing Prebiotic Animal Feed from Sugar Beet Pulp. Biomolecules.

[8] Dygas D., Kręgiel D., Berłowska J. (2023). Sugar Beet Pulp as a Biorefinery Substrate for Designing Feed. Molecules.

[9] Mebratu A.T., Vanhandsaeme L., Asfaw Y.T., Merckx W., Janssens G.P.J. (2023). Exploring fibrous ingredients for fish: The case of feeding sugar beet pulp to tambaquí *(Colossoma macropomum*). Heliyon.

[10] Badaras S., Klupsaite D., Ruzauskas M., Gruzauskas R., Zokaityte E., Starkute V., Mockus E., Klementaviciute J., Cernauskas D., Dauksiene A. (2022). Influence of Sugar Beet Pulp Supplementation on Pigs’ Health and Production Quality. Animals.

[11] Francis G., Makkar H.P.S., Becker K. (2001). Antinutritional factors present in plant-derived alternate fish feed ingredients and their effects in fish. Aquaculture.

[12] Alvarez A., Rodríguez A., Chaparro S., Borrás L.M., Rache L.Y., Brijaldo M.H., Martínez J.J. (2025). Solid-State Fermentation as a Biotechnological Tool to Reduce Antinutrients and Increase Nutritional Content in Legumes and Cereals for Animal Feed. Fermentation.

[13] Siddik M.A.B., Julien B.B., Islam S.M.M., Francis D.S. (2024). Fermentation in aquafeed processing: Achieving sustainability in feeds for global aquaculture production. Rev. Aquac..

[14] Dawood M.A.O., Koshio S. (2019). Application of fermentation strategy in aquafeed for sustainable aquaculture. Rev. Aquac..

[15] Ikusika O.O., Akinmoladun O.F., Mpendulo C.T. (2024). Enhancement of the Nutritional Composition and Antioxidant Activities of Fruit Pomaces and Agro-Industrial Byproducts through Solid-State Fermentation for Livestock Nutrition: A Review. Fermentation.

[16] Yafetto L., Odamtten G.T., Wiafe-Kwagyan M. (2023). Valorization of agro-industrial wastes into animal feed through microbial fermentation: A review of the global and Ghanaian case. Heliyon.

[17] El-Dakar A.Y., Elgamal A.A., Amer M.A.B., Mohammed A.S., Abdel-Aziz M.F. (2023). Evaluation of fermented soybean meal by *Bacillus subtilis* as an alternative to fishmeal on the growth, and physiological status of Nile tilapia *Oreochromis niloticus* fingerlings. Heliyon.

[18] Dharmakar P., Aanand S., Kumar J.S.S., Ande M.P., Padmavathy P., Pereira J.J. (2022). Fermented cottonseed meal as an alternative for groundnut oil cake in aquafeed. Int. J. Fish. Aquat. Stud..

[19] Wang T., Wang J., Zhang S., Xu J., Dong X., Miao S., Sun L. (2023). Effects of Solid-State Fermented (SSF) Pelleted Feed with *Lactobacillus plantarum* on *Tachysurus fulvidraco*: Growth, Digestion, Antioxidant, Immunity, Intestinal Morphology, and Microbiota. Fishes.

[20] Jiang Y., Zhao P.-F., Lin S.-M., Tang R.-J., Chen Y.-J., Luo L. (2018). Partial substitution of soybean meal with fermented soybean residue in diets for juvenile largemouth bass, *Micropterus salmoides*. Aquac. Nutr..

[21] Vieira L., Filipe D., Amaral D., Magalhães R., Martins N., Ferreira M., Ozorio R., Salgado J., Belo I., Oliva-Teles A. (2023). Solid-State Fermentation as Green Technology to Improve the Use of Plant Feedstuffs as Ingredients in Diets for European Sea Bass *(Dicentrarchus labrax*) Juveniles. Animals.

[22] Shi L., Xue M., Xu C., Jiang N., Fan Y., Chen J., Liu W., Wu Y., Zeng L., Zhou Y. (2023). Synergistic Impact of *Lactobacillus plantarum* and *Bacillus coagulans* on Solid-State Fermentation of *Astragalus* and Effects of Fermentation Products on Disease Resistance of Crucian Carp (*Carassius auratus*). Aquac. Res..

[23] Chi Z., Chi Z., Liu G., Wang F., Ju L., Zhang T. (2009). *Saccharomycopsis fibuligera* and its applications in biotechnology. Biotechnol. Adv..

[24] Chen L., Chi Z.M., Chi Z., Li M. (2010). Amylase production by *Saccharomycopsis fibuligera* A11 in solid-state fermentation for hydrolysis of Cassava starch. Appl. Biochem. Biotechnol..

[25] Ibrahim D., El-Sayed H.I., Mahmoud E.R., El-Rahman G.I.A., Bazeed S.M., Abdelwarith A.A., Elgamal A., Khalil S.S., Younis E.M., Kishawy A.T.Y. (2023). Impacts of Solid-State Fermented Barley with Fibrolytic Exogenous Enzymes on Feed Utilization, and Antioxidant Status of Broiler Chickens. Vet. Sci..

[26] Rodríguez-Estrada U., González-Alfaro K., Shene C. (2020). Replacement of Fish Meal by Solid State Fermented Lupin (*Lupinus albus*) Meal with *Latobacillus plantarum* 299v: Effect on Growth and Immune Status of Juvenile Atlantic Salmon (*Salmo salar*). Ann. Anim. Sci..

[27] Lian X., Shi M., Liang Y., Lin Q., Zhang L. (2024). The Effects of Unconventional Feed Fermentation on Intestinal Oxidative Stress in Animals. Antioxidants.

[28] Singh B., Kumar G., Kumar V., Singh D. (2021). Enhanced Phytase Production by *Bacillus subtilis* subsp. subtilis in Solid State Fermentation and its Utility in Improving Food Nutrition. Protein Pept. Lett..

[29] Qi N., Zhan X., Milmine J., Chang K.H., Li J. (2024). A novel thermophilic strain of *Bacillus subtilis* with antimicrobial activity and its potential application in solid-state fermentation of soybean meal. Microbiol. Spectr..

[30] Huang H., Li X., Guo B., Zhang Y., Yang X., Liu Y., Leng X. (2024). The Substitution of Fishmeal with Yeast Culture in the Yellow Catfish *(Pelteobagrus fulvidraco*) Diet: Growth, Serum Biochemical Indices, and Intestinal and Hepatopancreatic Histology. Animals.

[31] (2002). Animal Feeding Stuffs — Sampling.

[32] Tang J., Lin B., Jiang W., Li Q., Zhu L., Zhang G., Chen Q., Yang Q., Yang S., Chen S. (2022). Screening of β -damascenone-producing strains in light-flavor *Baijiu* and its production optimization via response surface methodology. Front. Microbiol..

[33] Wu Q., Li M., Bilal M., Yang Y., Zhang J., Li X. (2022). Enhanced Production of Mycophenolic Acid from *Penicillium brevicompactum* via Optimized Fermentation Strategy. Appl. Biochem. Biotechnol..

[34] AOAC (2005). Official Methods of Analysis of the AOAC International.

[35] Soest P.J.V., Robertson J.B., Lewis B.A. (1991). Methods for dietary fiber, neutral detergent fiber, and nonstarch polysaccharides in relation to animal nutrition. J. Dairy Sci..

[36] Dai L., Yang L., Wang Y., Li Y., Zhao J., Pan S., Li Y., Yang D., He D. (2024). An Optimized Microwave-Assisted Digestion Method to Analyze the Amino Acids Profile of Quisqualis Fructus from Different Planted Origins. Foods.

[37] Arnetoli M., Montegrossi G., Buccianti A., Gonnelli C. (2008). Determination of organic acids in plants of *Silene paradoxa* L. by HPLC. J. Agric. Food Chem..

[38] Liao H., Liu P., Deng Y., Zhang W., Pan C., Jia Y., Long F., Tang H. (2022). Feeding effects of low-level fish meal replacement by algal meals of *Schizochytrium limacinum* and *Nannochloropsis salina* on largemouth bass (*Micropterus salmoides*). Aquaculture.

[39] Jiang W., Zhang Y., Yuan M., Liu Y., Deng J., Tan B. (2022). Effects of different types of non-starch polysaccharides on growth, digestive enzyme activity, intestinal barrier function and antioxidant activity of tilapia (*Oreochromis niloticus*). Aquac. Rep..

[40] Shen J., Liu H., Tan B., Dong X., Yang Q., Chi S., Zhang S. (2020). Effects of replacement of fishmeal with cottonseed protein concentrate on the growth, intestinal microflora, haematological and antioxidant indices of juvenile golden pompano *(Trachinotus ovatus*). Aquac. Nutr..

[41] Fan J., Zhang Y., Zhou H., Liu Y., Cao Y., Dou X., Fu X., Deng J., Tan B. (2023). Dietary Malondialdehyde Damage to the Growth Performance and Digestive Function of Hybrid Grouper *(Epinephelus fuscoguttatus* ♀ × *E. lanceolatu* ♂). Animals.

[42] Zhao W., Xie J.-J., Fang H.-H., Liu Y.-J., Tian L.-X., Niu J. (2020). Effects of corn starch level on growth performance, antioxidant capacity, gut morphology and intestinal microflora of juvenile golden pompano, *Trachinotus ovatus*. Aquaculture.

[43] Buamard N., Benjakul S. (2018). Combination effect of high pressure treatment and ethanolic extract from coconut husk on gel properties of sardine surimi. LWT-Food Sci. Technol..

[44] Korkmaz K. (2023). The Effect of Sodium Bicarbonate Injection on the Physico-Chemical Quality of Post-Harvest Trout. Foods.

[45] Niu F.C., Huang Y.H., Cao J.M., Wang G.X., Sun Y.P., Zhao H.X., Li Y.J., Ma Y.P. (2015). Effects of five additives on growth performance, body composition and serum biochemical indices of yellow catfish (*Pelteobagrus fulvidraco*). Dongwu Yingyang Xuebao.

[46] Shi Y., Zhong L., Ma X., Liu Y., Tang T., Hu Y. (2019). Effect of replacing fishmeal with stickwater hydrolysate on the growth, serum biochemical indexes, immune indexes, intestinal histology and microbiota of rice field eel (*Monopterus albus*). Aquac. Rep..

[47] Zhang M., Wang S., Qin C., Li M. (2023). Dietary inulin benefits on growth, digestive ability and intestinal microbiota in yellow catfish exposed to ammonia. Aquac. Rep..

[48] Sheng Z., Xu J., Zhang Y., Wang Z., Chen N., Li S. (2022). Dietary protein hydrolysate effects on growth, digestive enzymes activity, and expression of genes related to amino acid transport and metabolism of larval snakehead (*Channa argus*). Aquaculture.

[49] Bitla A.R., Kumari N.M., Reddy N.S., Nagaraju K.V., Sachan A., Kumar V.P., Suchitra M.M., Srinivasa Rao P.V.L.N. (2012). Antioxidant status in patients with metabolic syndrome as measured by ferric reducing ability of plasma (FRAP) assay. J. Clin. Sci. Res..

[50] Benzie I.F.F., Strain J.J. (1996). The ferric reducing ability of plasma (FRAP) as a measure of “antioxidant power”: The FRAP assay. Anal. Biochem..

[51] Moretti D., Nordi W., Cruz T., Machado R. (2017). Catalase, superoxide dismutase, glutathione peroxidase and oxygen radical absorbance capacity in the gut of juvenile pacu *Piaractus mesopotamicus* and dourado Salminus brasiliensis fed bovine first milk secretion. Lat. Am. J. Aquat. Res..

[52] Zhao W., Liu Z.-L., Niu J. (2020). Growth performance, intestinal histomorphology, body composition, hematological and antioxidant parameters of Oncorhynchus mykiss were not detrimentally affected by replacement of fish meal with concentrated dephenolization cottonseed protein. Aquac. Rep..

[53] Feng X.Y., Ng K., Ajlouni S., Zhang P.Z., Liang Z.J., Fang Z.X. (2025). Enhancement of Protein Hydrolysis and Bioactivity in Hempseed Cake via Solid-State Fermentation Using Aspergillus niger, *Bacillus subtilis*, and Lactobacillus rhamnosus. Food Bioprocess Technol..

[54] Wang Y., Liu J., Wei F.H., Liu X.L., Yi C.X., Zhang Y.G. (2019). Improvement of the nutritional value, sensory properties and bioavailability of rapeseed meal fermented with mixed microorganisms. LWT-Food Sci. Technol..

[55] Salamanca N., Herrera M., de la Roca E. (2025). Amino Acids as Dietary Additives for Enhancing Fish Welfare in Aquaculture. Animals.

[56] Hassaan M.S., Soltan M.A., Mohammady E.Y., Elashry M.A., El-Haroun E.R., Davies S.J. (2018). Growth and physiological responses of Nile tilapia, *Oreochromis niloticus* fed dietary fermented sunflower meal inoculated with Saccharomyces cerevisiae and *Bacillus subtilis*. Aquaculture.

[57] Chizhayeva A., Amangeldi A., Oleinikova Y., Alybaeva A., Sadanov A. (2022). Lactic acid bacteria as probiotics in sustainable development of aquaculture. Aquat. Living Resour..

[58] Alhamadany A.S., Mohamed M.J., Al-Gharawi J.K. (2023). Synergistic Effect of Butyric and Propionic Supplementation in Common Carp Diets on Growth and Immunological Parameters. IOP Conf. Ser. Earth Environ. Sci..

[59] El-Sayed A.-F.M., Eissa E.-S.H., Hendam B.M., Dighiesh H.S., Elnabi H.E.A., El-Aziz Y.M.A., Eissa M.E.H., Ghanem S.F. (2025). Dietary organic acid blend modulates hemato-immunological parameters, digestive and reproductive performances in red tilapia (*Oreochromis niloticus* × *O. mossambicus*) broodstock. Fish Physiol. Biochem..

[60] Mohammady E.Y., Aboseif A.M., Al-Afify A.D., Abdelhameed M.S., Shawer E.E., Abdo S.M., Ramadan E.A., Hegab M.H., El-Dein A.N., Hassaan M.S. (2024). Growth and physiological responses of Nile tilapia, *Oreochromis niloticus* fed dietary fermented sugar beet bagasse and reared in biofloc system. Anim. Feed Sci. Technol..

[61] Curry S.M., Blavi L., Wiseman J., Stein H.H. (2019). Effects of distillers dried grains with solubles on amino acid digestibility, growth performance, and carcass characteristics of growing pigs. Transl. Anim. Sci..

[62] Liu C., Li Y.L., Sun W.M., Ma F.Y., Wang X.W., Yang Z.H. (2025). New Techniques of Meat Quality Assessment for Detecting Meat Texture. Processes.

[63] Wang M.Q., Feng L., Wu P., Liu Y., Ren H.M., Jin X.W., Zhou X.Q., Jiang W.D. (2025). Regulation of muscle springiness and hardness: The role of myo-inositol in enhancing fish flesh texture. Food Chem.-X.

[64] Sarmiento P., Castro P.L., Ginés R. (2025). Impact of Alternative Feed Ingredients and Feeding Strategies on Growth, Muscle Morphology, and Fillet Quality of Genetically Selected Gilthead Seabream (*Sparus aurata*) in a Long-Term Feeding Trial. Animals.

[65] Dawood M.A.O., Eweedah N.M., Khalafalla M.M., Khalid A. (2020). Evaluation of fermented date palm seed meal with Aspergillus oryzae on the growth, digestion capacity and immune response of Nile tilapia (*Oreochromis niloticus*). Aquac. Nutr..

[66] Nasr M.A.F., Reda R.M., Ismail T.A., Moustafa A. (2021). Growth, Hemato-Biochemical Parameters, Body Composition, and Myostatin Gene Expression of Clarias gariepinus Fed by Replacing Fishmeal with Plant Protein. Animals.

[67] Tang S., Ma H., Hua X., Wang L., Yun B., Zhu X., Qian X. (2025). Effects of Fish Meal Replacement with Poultry By-Product Meal on Growth Performance, Lipid Metabolism, Hepatic–Intestinal Health and Ammonia Nitrogen Stress in Siniperca chuatsi. Fishes.

[68] Langkilde A.M., Andersson H., Bosaeus I. (1993). Sugar-beet fibre increases cholesterol and reduces bile acid excretion from the small bowel. Br. J. Nutr..

[69] Marques A., Balen R.E., Fernandes L.D.P., Motta C.M., de Assis H.C.S., Taher D.M., Meurer F., Vargas J.V.C., Mariano A.B., Cestari M.M. (2019). Diets containing residual microalgae biomass protect fishes against oxidative stress and DNA damage. J. Appl. Phycol..

[70] Ming J.-H., Ye J.-Y., Zhang Y.-X., Xu P., Xie J. (2015). Effects of dietary reduced glutathione on growth performance, non-specific immunity, antioxidant capacity and expression levels of IGF-I and HSP70 mRNA of grass carp (*Ctenopharyngodon idella*). Aquaculture.

[71] Maria do Carmo de Carvalho e M., Amanda Suellenn da Silva Santos O., Liriane Andressa Alves da S., Maísa Guimarães Silva P., Vanessa Brito de Carvalho L., Patel V.B., Preedy V.R. (2022). Biological Indicators of Oxidative Stress [Malondialdehyde, Catalase, Glutathione Peroxidase, and Superoxide Dismutase] and Their Application in Nutrition. Biomarkers in Nutrition.

[72] Dossou S., Koshio S., Ishikawa M., Yokoyama S., Dawood M.A., El Basuini M.F., El-Hais A.M., Olivier A. (2018). Effect of partial replacement of fish meal by fermented rapeseed meal on growth, immune response and oxidative condition of red sea bream juvenile, *Pagrus major*. Aquaculture.

[73] Suzer C., Çoban D., Kamaci H.O., Saka Ş., Firat K., Otgucuoğlu Ö., Küçüksari H. (2008). *Lactobacillus* spp. bacteria as probiotics in gilthead sea bream (*Sparus aurata*, L.) larvae: Effects on growth performance and digestive enzyme activities. Aquaculture.

[74] Hidalgo M.C., Urea E., Alberto S.-C. (1999). Comparative study of digestive enzymes in fish with different nutritional habits. Proteolytic and amylase activities. Aquaculture.

[75] Ayiku S., Shen J.-F., Tan B.-P., Dong X.-H., Liu H.-Y. (2020). Effects of dietary yeast culture on shrimp growth, immune response, intestinal health and disease resistance against Vibrio harveyi. Fish Shellfish Immunol..

[76] Yang H., Bian Y., Huang L., Lan Q., Ma L., Li X., Leng X. (2022). Effects of replacing fish meal with fermented soybean meal on the growth performance, intestinal microbiota, morphology and disease resistance of largemouth bass (*Micropterus salmoides*). Aquac. Rep..

[77] Takahashi L.S., Biller-Takahashi J.D., Mansano C.F.M., Urbinati E.C., Gimbo R.Y., Saita M.V. (2016). Long-term organic selenium supplementation overcomes the trade-off between immune and antioxidant systems in pacu (*Piaractus mesopotamicus*). Fish Shellfish Immunol..

[78] Mansour A.T., Esteban M.Á. (2017). Effects of carbon sources and plant protein levels in a biofloc system on growth performance, and the immune and antioxidant status of Nile tilapia (*Oreochromis niloticus*). Fish Shellfish Immunol..

[79] Tan X., Sun Z., Zhou C., Huang Z., Tan L., Xun P., Huang Q., Lin H., Ye C., Wang A. (2018). Effects of dietary dandelion extract on intestinal morphology, antioxidant status, immune function and physical barrier function of juvenile golden pompano *Trachinotus ovatus*. Fish Shellfish Immunol..

[80] Morales M., Munné-Bosch S. (2019). Malondialdehyde: Facts and Artifacts. Plant Physiol..

[81] Lin Y.-H., Chen Y.-T. (2021). *Lactobacillus* spp. fermented soybean meal partially substitution to fish meal enhances innate immune responses and nutrient digestibility of white shrimp (*Litopenaeus vannamei*) fed diet with low fish meal. Aquaculture.

[82] Zhang M., Sun Y., Chen K., Yu N., Zhou Z., Chen L., Du Z., Li E. (2014). Characterization of the intestinal microbiota in Pacific white shrimp, *Litopenaeus vannamei*, fed diets with different lipid sources. Aquaculture.

[83] Ahmed R.O., Ali A., Leeds T., Salem M. (2023). Fecal Microbiome Analysis Distinguishes Bacterial Taxa Biomarkers Associated with Red Fillet Color in Rainbow Trout. Microorganisms.

[84] Jiang Y.-H., Liang M., Yang Y.-H., Xue J., Suo H.-Y. (2024). Probiotic *Lactobacillus plantarum* SHY21-2 protected zebrafish against Aeromonas hydrophila infection by maintaining intestinal barrier integrity, inhibiting inflammatory and oxidative stress responses, and regulating intestinal microbiome. Aquaculture.

[85] Qi X., Zhang Y., Zhang Y., Luo F., Song K., Wang G., Ling F. (2023). Vitamin B12 produced by *Cetobacterium somerae* improves host resistance against pathogen infection through strengthening the interactions within gut microbiota. Microbiome.

[86] Wang S., Li X., Zhang M., Li M. (2025). Administration of *Cetobacterium somerae* ceto isolated from the intestine of yellow catfish (*Pelteobagrus fulvidraco*) as potential probiotics against chronic ammonia stress. Aquaculture.

