# Supplementary material for: Optimized Solid-State Fermentation of Sugar Beet Pulp with Mixed Microbes Improves Its Nutritional Value and Promotes Growth, Health, and Intestinal Function in Yellow Catfish (Pelteobagrus fulvidraco)
Source: Animals (Basel). 2026 Mar 14;16(6):915. doi: 10.3390/ani16060915 (PMC13023332; doi:10.3390/ani16060915)
Supplement: Supplementary file 1 [file animals-16-00915-s001.zip › animals-4150121-supplementary.pdf]

Supplemental Figures

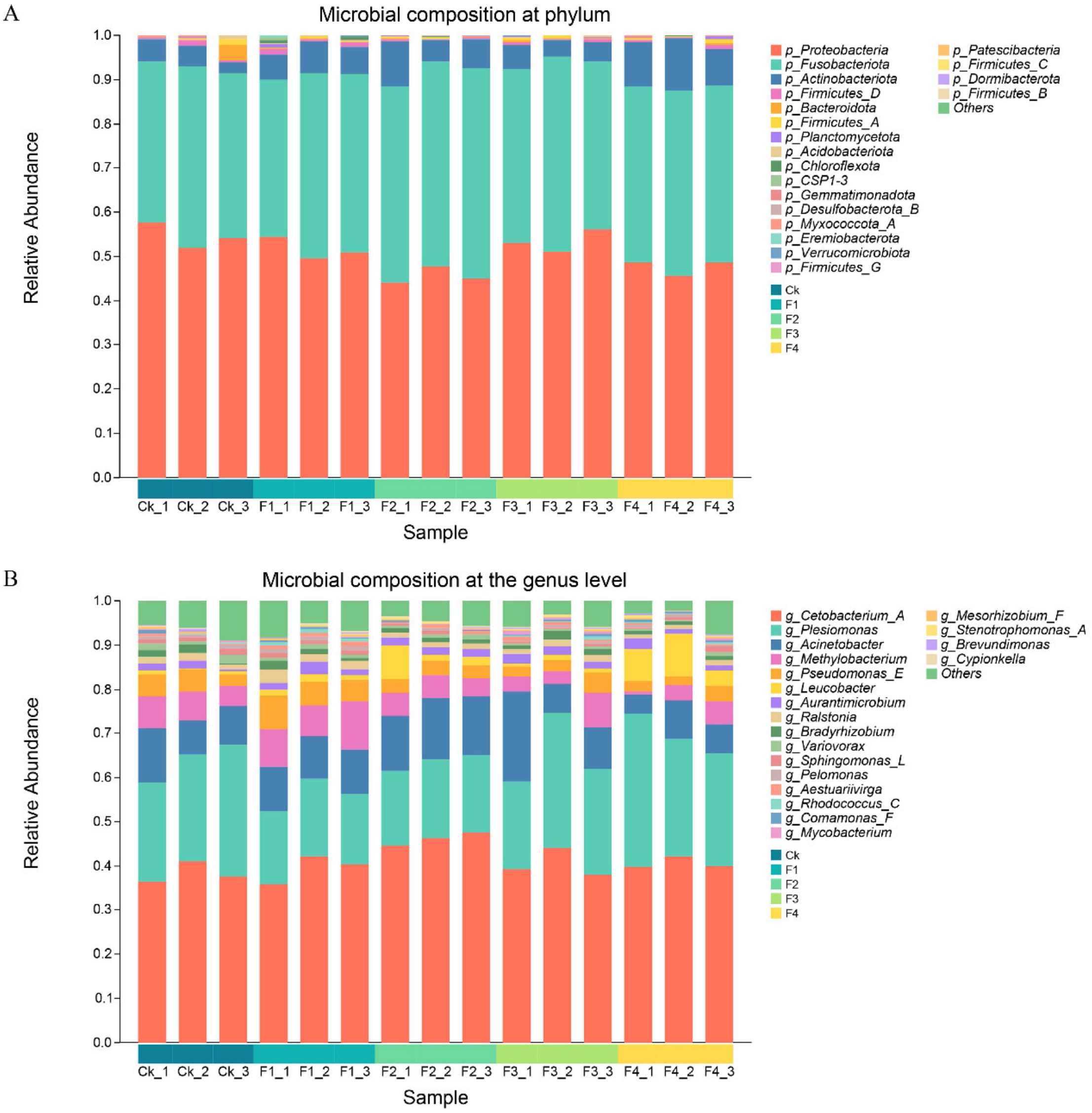

Figure S1. Intestinal microbial community composition of juvenile yellow catfish (Pelteobagrus fulvidraco) at the phylum and genus levels following dietary fermented sugar beet pulp (FBP) supplementation.

(A) Relative abundance of intestinal microbiota at the phylum level; (B) Relative abundance of intestinal microbiota at the genus level. Only dominant taxa with relative abundance >0.01 are displayed in both panels, and all low-abundance taxa with relative abundance ≤0.01 are pooled as "Others" for clarity. Experimental groups include CK (0% FBP, control), F1 (3% FBP), F2 (6% FBP), F3 (9% FBP), and F4 (12% FBP), with 3 biological replicates per group (labeled as 1, 2, 3 for each group on the x-axis).

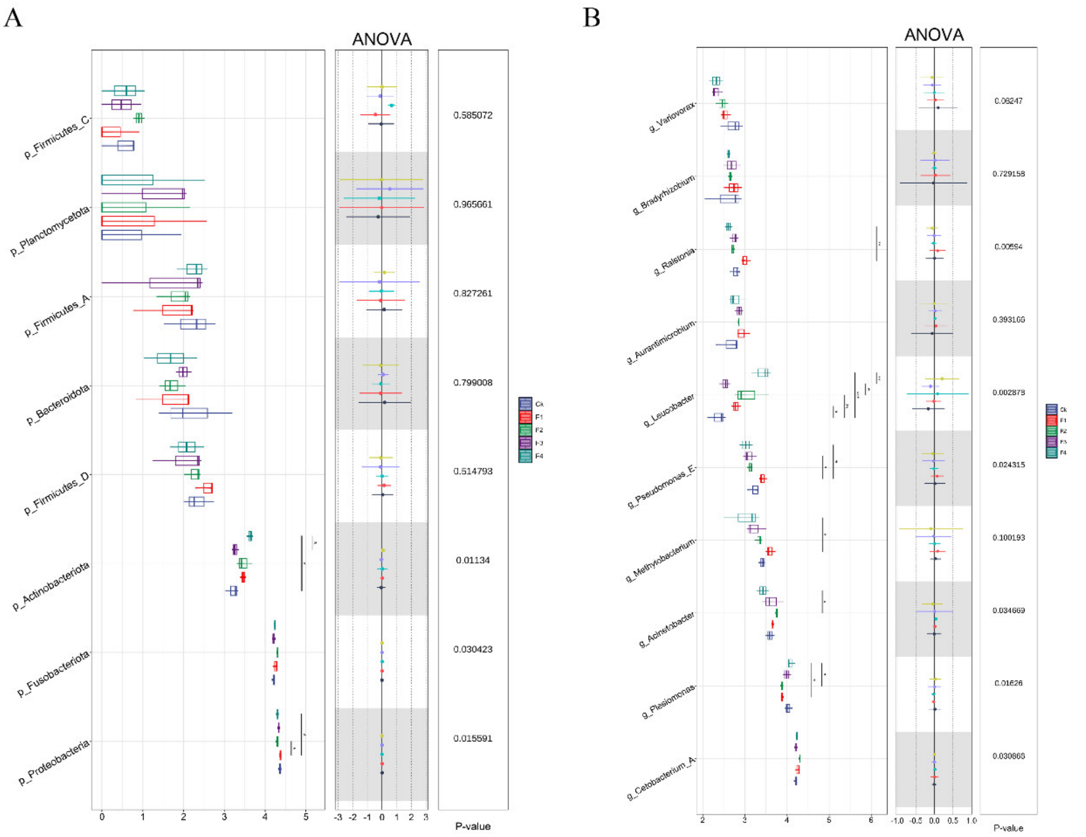

Figure S2. Differentially abundant intestinal microbial taxa in juvenile yellow catfish (*Pelteobagrus fulvidraco*) fed diets with different fermented sugar beet pulp (FBP) inclusion levels. (A) Analysis of differentially abundant intestinal microbial taxa at the phylum level; (B) Analysis of differentially abundant intestinal microbial taxa at the genus level. Experimental groups: CK (0% FBP, control), F1 (3% FBP), F2 (6% FBP), F3 (9% FBP), F4 (12% FBP), with three biological replicates per group. Intergroup differences in microbial relative abundance were statistically analyzed by one-way ANOVA followed by the Conover-Iman post-hoc test, where $P < 0.05$ was considered significantly different (*), $P < 0.01$ was considered extremely significantly different (**), and $P < 0.001$ was considered highly extremely significantly different (***). The corresponding ANOVA P-values for each taxon are shown on the right side of the figure.
